# DNA methylation across the tree of life, from micro to macro-organism

**DOI:** 10.1080/21655979.2021.2014387

**Published:** 2022-01-06

**Authors:** Nasrullah, Abrar Hussain, Sagheer Ahmed, Mahmood Rasool, Abdul Jabbar Shah

**Affiliations:** a Center for Advanced Studies in Vaccinology & Biotechnology (Casvab), University of Baluchistan, Quetta- Pakistan. E-mails: nasrullahkakar007@gmail.com; b Department of Biotechnology, Faculty of Life Sciences, Buitems, Quetta-Pakistan. E-mails: Abrarbangash176@hotmail.com; c Department of Basic Medical Sciences, Shifa College of Pharmaceutical Sciences, Shifa Tameer-e-Millat University, Islamabad, Pakistan. E-mails: Sagheer.scps@stmu.edu.pk; d Center of Excellence in Genomic Medicine Research, Department of Medical Laboratory Technology, Faculty of Applied Medical Sciences, King Abdulaziz University, Jeddah, Saudi Arabia. E-mails: Mahmoodrasool@yahoo.com; e Department of Pharmaceutical Sciences, Comsats University, Abbottabad. E-mails: Jabbarshah@cuiatd.edu.pk

**Keywords:** DNA methylation, methyltransferases, epigenetics, CpG

## Abstract

DNA methylation is a process in which methyl (CH3) groups are added to the DNA molecule. The DNA segment does not change in the sequence, but DNA methylation could alter the action of DNA. Different enzymes like DNA methyltransferases (DNMTs) take part in methylation of cytosine/adenine nucleosides in DNA. In prokaryotes, DNA methylation is performed to prevent the attack of phage and also plays a role in the chromosome replication and repair. In fungi, DNA methylation is studied to see the transcriptional changes, as in insects, the DNA methylation is not that well-known, it plays a different role like other organisms. In mammals, the DNA methylation is related to different types of cancers and plays the most important role in the placental development and abnormal DNA methylation connected with diseases like cancer, autoimmune diseases, and rheumatoid arthritis.

## Graphical abstract

**Figure:**
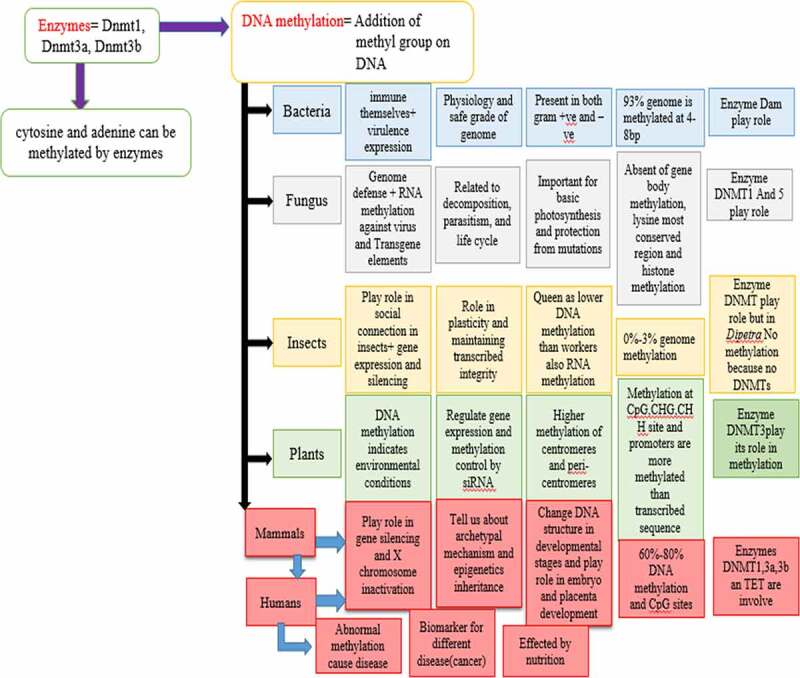

## Introduction

In the process of DNA methylation, methyl groups are added to the base pairs cytosine and adenine at the fifth position of the nucleoside in double-stranded DNA, at different sites like CpG, CHG, and CHH, DNA methylation takes place, and by using this process, the DNA molecule activity is changed easily without interrupting its sequence [1]. The main methylation process widely used in prokaryotes and eukaryotes is the methylation of 5-methylcytosine. Two of the DNA’s four bases cytosine and adenine can be methylated. Its main importance is in the study of gene regulation, gene silencing, gene activation, and many other functions like cell differentiation [2]. The catalyzation of this process is done by the enzymes methyltransferases (Dnmt1, Dnmt3a, and the third one is Dnmt3b), which have different functions.

Two ways of DNA demethylation are as follows:

1. An enzymatic process, which leads to the removal of the methyl group from 5mC, is called DNA demethylation.
2. In opposite passive DNA demethylation is the one which lack maintain methylation throughout successive DNA replication either in the absence of Dnmt1 or because of its inhibition and patterns in methylation are *overall methylation patterns Methylation of specific genes, tissue-specific methylation pattern*. DNA markers are introduced by these methylase enzymes, which give hints for a diversity of processes that include physiological processes of cell cycle and gene expression in epigenetics control in bacteria and another organism [3]. DNA methylation plays a significant role in the physiology and is examined in two methyltransferase models, from *Alphaproteo bacteria CcrM methylase* and from Gamma *proteo bacteria Dam methylase* [4–7].

**Graph.1;:**
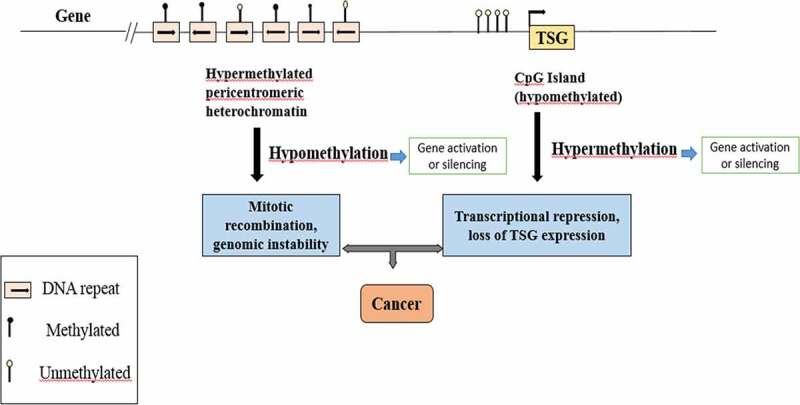
The diagram shows a representative region of genomic DNA in a normal cell. The region shown contains repeat-rich, hypermethylated pericentromeric heterochromatin and an actively transcribed tumor suppressor gene (TSG) associated with a hypomethylated CpG Island (indicated in red). In tumor cells, repeat-rich heterochromatin becomes hypomethylated and this contributes to genomic instability, a hallmark of tumor cells, through increased mitotic recombination events. De novo methylation of CpG Islands also occurs in cancer cells and can result in the transcriptional silencing of growth-regulatory genes. These changes in methylation are early events in tumorigenesis.

Due to DNA methylation, the chromatin structure can undergo changes directly and prevent transcription factor binding and methylation plays an important role in numerous cellular processes and components, including genomic imprinting, embryonic development, preservation of chromosome stability, and X-chromosome inactivation. DNA methylation also regulates transcription, chromosome replication, repair, and most possibly additional important processes. However, some motifs and DNA molecules are 100% methylated, while some motifs are nonmethylated [8–11]. The researcher went through 230 species of bacterial and archaeal origin having varying genomes and got the evidence that 93% of genomes are methylated, while in fungi, the genome is methylated by DNA methyltransferase 1 (DNMT1) [12] and DNA methyltransferase 5 (DNMT5) [13] with unknown biological characteristics and methylation of cytosine (5-methylcytosine) 5mC has been concerned in defense of genome. By investigating insects’ DNA, methylation is mostly directed to broadly expressed genes [14,15]. But certain sets of insects have totally missing useful DNA methylation schemes and counting model dipterans [14]. In insects, it is related to plasticity and regulates the phenotype of insects [16–21]. Keeping in mind the global sustainability development goals to protect, restore, and use the sustainable use of ecosystem in which fungi and plants are major stock holder, we focus on both fungi and plants [22]. In plants, animals, and fungi, the DNA methylation of histone at the nucleotide lysine is the most conserved marker that is correlated with the function of gene activation and gene silencing. In animals, 5mC is formed due to insertion or deletion of the methyl group in CG sites (CpG) due to which gene regulation is initiated [23,24]. All the sites methylated in the humans are done on the somatic cells [25,26].

## DNA methylation in Bacteria:

DNA methylation is utilized as a basic tool for different studies in bacteria to know the functions of genes and their restriction/modification system [27–29] by selective degradation of exogenous DNA of bacteriophage. Bacterial species immune themselves through the use of type II restriction-modification (R-M) systems, and different groups of prokaryotes immune their DNA from phage or extracellular DNA through methylation by sequence-specific restriction-modification (R-M) systems. In this system, DNA methyltransferases add the methyl group in bacterial DNA exactly at 4–8 base pairs (b.p.) and unmethylated DNA is degraded [30]. Several other functions such as transcription, repair, chromosome replication, and most possible other essential procedures are regulated through that system. The regulation of transcription of few genes [31–33] and chromosome replication [5,34] are regulated by Dam (DNA adenine methyltransferase) methylation in bacteria at GATC sequences (sequence at which methylation occurs). All GATC sequences of DNA are methylated at the 6^th^ position of the adenine base in *Escherichia coli*, and this process is called dam methylation; for the role of dam methylation, one hypothesis is that mismatch repair enzymes are used to distinguish among new and old strands of DNA; at the replication fork, this process is followed by DNA methylation. To support this hypothesis, at the unmethylated strand of the heteroduplex DNA, mismatched bases are preferentially removed [35,36]. DNA adenine methyltransferase (Dam) methylates DNA in *Escherichia coli* at the adenine residue. Cellular functions such as DNA mismatch repair, gene transcription, nucleoid structure, and initiation of chromosome replication are influenced by the level of Dam and these methylated residues. *Caulobacter crescentus* CcrM enzymes and *Escherichia coli Dam* are the two best studied DNA methyltransferases. *Caulobacter crescentus* CcrM (cell cycle-regulated methyltrasferase) and *Escherichia coli* Dam and enzymes do not apparently relate to a restriction enzyme, which are the two best studied DNA methyltransferases.

In kingdoms bacteria and archaea, N^6^-methyl adenine (6 mA) has been found as a dynamic DNA epigenetic modification [37]. *N*^6^-methyldeoxyadenosine (6 mA) is a type of DNA methylation, which has functional roles in living organisms, and DAMT1 is involved in it. Adenine methylation is catalyzed by DNA-adenine methyltransferase that adds the methyl group in adenine at the sixth position of the purine ring [38,39]. In bacteria, virulence gene expression is directly controlled by DNA adenine methylation [31,40]. Among the bacteria, some Gram-negative and Gram-positive organisms perform the phenomenon of DNA methylation. The best characterized orphan methyltransferase apart from Dam is CcrM. It is a necessary DNA methyltransferase of an aquatic bacterium with dimorphic nature. Both Dam and CcrM have the ability to catalyze the transmission of the methyl group from S-adenosylmethionin to the N-6 position of the adenine at a definite target sequence. However, they fit into distinct methyltransferase groups. There is also a difference in their target sequence such as GANTC for CcrM and GATC for DAM. In *Escherichia coli*, about 50 years ago, restriction modification (R-M) systems were first identified and are now known to be abundant among other bacterial species. Generally, two distinct enzyme activities are included in R-M systems: first, specific restriction endonuclease enzyme that cuts the DNA at a particular recoganization sequence and second, a transferase enzyme named DNA methyltransferase has the ability to methylate the DNA at the same particular sites. So DNA methyltransferase enzyme prevents the cleavage of DNA by the associated restriction enzyme. The type II R-M system includes two distinct types of proteins. These proteins possess independent enzymatic actions, endonucleases activity by restriction endonuclease enzyme, and DNA transferase activity by DNA methyl transferase [41]. Not only the DNA methylation occurs, but also demethylase enzymes are catalyzed by the reversible modification (demethylation) [42]. In bacteria, the methylation process works in bacterial physiology, making the bacteria safe from all other foreign antibacterial organisms like phages and R-M system.

### (a) Role of DNA cytosine methylation in bacterial physiology

In bacterial genomics, T:G mismatches appear spontaneously due to deamination of C5-methylcytosine that creates thymine. The repair system in enteric bacteria precisely restores such mismatches, and it clearly appears for the establishment of the tricky side of C5-methylcytosine [43]. An assumption was made that establishment of T:G mismatches could be a settlement for unidentified physiological paybacks of m5C. On the other hand, at least under laboratory conditions, phenotypic consequences in *E. coli* did not seem due to loss of the lonely methyltransferase Dcm [44].

But recent studies propose that regulation of gene expression is a physiological role that may be due to DNA cytosine methylation. In *H. pylori*, gene expression, which was involved in adhesion, motility, and virulence, changes in the absence of an orphan C5-methylcytosine methyltransferase also called Hpy A – VIBM [45], and in *E.coli,* expression of the stress response sigma factor Rpo S has increased in the absence of DNA cytosine methylation [46]. Overexpression of a membrane transporter involves the ethidium bromide transporter found in *E. coli* dcm mutants [47].

## DNA methylation in Fungus

As camper to other eukaryotes, DNA methylation is less studied and less information is available about it in fungi. In the *Uncinocarpus reesii*, the DNA methylation is present in the noncoding region and in the coding region. It gives rise to the consensus that DNA methylation is initially dedicated to transposable elements (TEs), which are supposed to maintain the integrity of DNA in fungi [12,48], and there is negative correlation between the TE methylation level and TE expression as the more the TE methylation, the less the TE expression and vice versa. The DNA methylation of CpG positions plays a vital role in the conservation and strength of methylation designs, in usual and pathological circumstances, displaying that the DNA methylation position is extremely dependent on the native order CpG. This connection examines the DNA sequence related to methylation shapes in both pathological and physiological environments of fungus.

Currently, many research papers have concluded that different amounts of DNA methylation in different fungal species are present, according to which 0.22% DNA is methylated in *Magnaporthe oryzae* [49], 0.38% to 0.42% genome is methylated in *Metarhizium robertsii* [50], 1.8% in *Ganoderma sinense* [51], 6.4%-7.7% genome is methylated in *Cryphonectria parasitica* [52], and 36.9%-39.6% methylation of genome in *Tuber melanosporum* [53]. More recent research showed that in fungi, the DNA methylation is around the genes [49–52], which have more complex function than we have observed in the past; they are also present in more complicated and divergent patterns, for example, they show their role in defense of genome like in bacteria [49,51,53], and also, research showed that they have dynamic epigenetic entity, play their role in the development of *M.oryzea* [49,50], and play role in the secondary metabolism and regulation of *G. sinense* [51] and cell morphology delay in *Candida Albicans* [54] by changing the transcriptional activities of related genes. DNA methylation is related to different lifestyles for example Decomposition, parasitism and life cycles of fungi, in *H, parvipoerum* 96,026 it is related to asexual (mycelia and conidia) lifestyle which was confirmed by Zhen Zeng et al in *H, parvipoerum* that DNA methylation have a role in the transcriptional regulation of asexual patterns, and *N.carsaa* before going to sexual cycle the spore fuses and before nuclear fusion take place a defense system is activated which works to prevent the repetitive sequencing such as TEs [55].

### (a) Fungal histone methylation distribution

In fungus, many novel enzymes are discovered, which can be easily studied because of their wide distribution, and also due to their high throughput sequencing, we came to know that most of the KMT genes in the kingdom mycota have the H3K4, H3K27, and H3K36 methylation; according to early capability, H3 potential lysine methylation cannot be absorbed in extant fungi [56].

Methylation of histone is widespread among the *hemiasco mycetes*, best model organism for the understanding is *S. cerevisiae* don’t have H3K9, H3K27 and cytosine methylation, another model organism *S. pombe* lack H3K27 and methylation of histone but the specie *Candida albicans* don’t have H3K9 and H3K27 but they might have cytosine DNA methylation [54].

In 2014, the DNA methyltransferase (DNMT5) was described, which methylates DNA in the *basidiomycete* a human pathogen *C. neoforman* [13]. The genes that code for DNMT5 enzymes are distributed widely in the genome of fungi, which work as cytosine methylation, and new development and high-efficiency sequencing reveal the adenine methylation in the fungi; it is believed that adenine methylation is not present in fungi [56]. In fungi, the loss of H3K27 methylation in a group with the loss of cytosine DNA is common and by comparison of structural SET domains, we came to know that no associations among lifestyle and ecology have occurred [57].

In the future, research has to be carried out on the poorly understood taxa of fungi like *chyrids* and *zygomycetes* to know the histone modification and DNA methylation and process of these as they are very important and connected to plant and animals as well [56]. Li et al. reveal that in *C. parasitica*, the influence of DNA methylation alters expression gene, as the RNA sequence shows that where genes are expressed lower and have higher methylation in the promoter region, DNA methylation takes place in the promoter and has a negative effect on gene expression [58].

### (b) Established gene body methylation is absent in fungi

In fungal genome, 5mC is not uniformly dispersed and 5mC occurs inside coding areas of the fungal genome, which is in divergence with CG DNA methylation enhancement in coding sequences of few species of insects and angiosperms as well and extremely preserved and expressed constitutively in some genes (gene body methylation) [12,59–62]. In species like *Uncinocarpus reesii*, a similar improvement has been observed at CH frameworks [12]. This was confirmed in species *U. reesii* and will be continued in further species (*A. bisporus, P. destructans,* and *C. cinerea*) with confirmation of upgrading with the CG situation spending method of an enrichment used by these [59,63].

It was revealed that 5mC is need not be narrowed to coding areas. However, they showed that genes with 5mC DNA methylation that crossed genes and intergenic sequence enrichment stood localized, also that genes that are CG-enriched in fungi do not display the similar normal-like spreading of CG methylation as in plants' gene body and few insect species and in fungal species genome through investigation 'by Bewick et al., we came to know that usually 5mC is narrow (<1.0%) and a minor amount of genes contributes to the bulk of 5mC stages, which is why these are indications that in fungi species, canonical gene body methylation is absent [61]. The genome methylation of fungi is very important for its basic photosynthetic ability and protection from different types of mutation and genome stability.

### (c) RNA methylation in fungi and DNA methylation in S. pombe, Aspergillus, and S. cerevisiae

RNA-methylated gene silencing is newly discovered that suppresses the gene expression at the posttranscriptional level also known as RNA silencing or as an intrinsic defense mechanism against viruses, transgenes, and transposable elements [64]. Double-stranded RNA regulates the degradation of homologous RNA by diminishing the gene expression [65]. DNA methylation is necessary for normal development and growth of either mammals or plants and other organisms, but DNA methylation totally absent in some organisms like Saccharomyces cerevisiae and there is little DNA methylation as in *Aspergillus* [66].

## DNA methylation in insects

In insects, the occurrence of 5-methylcytosine has been testified in many species. The DNA methylation is not always present in insects, and some species also show the absence of methylation and those that have DNA methylation need to be understood with great care since the methylation can be confined to the specific development stage to particular sequences and the DNA methylation is preserved in most species if not in all. In insects, DNA methylation is still poorly recognized as we do not have such a good model organism to understand insects’ DNA methylation function'; according to data, insects have a variable number of DNA methylations, which might have variable factions [67]. In the insects, the methylation of the gene body was very much considered [14].

Investigational facts have revealed that the stages of methylation of gene body of the honey bee and silk moth are considerably inferior associated with further invertebrates (sea anemone, Ciona intestinally, *Nematostella vectensis,* and sea squirt) [68]. All these results have supported the assumption that in insects, ancestor DNA methylation was decreased in *Drosophila melanogaster* [69], Fruit fly, and *Tribolium castaneum*, flour beetles; like model organisms, insects donot have notable DNA methylation level in their genome [12,70]. On the other hand, in honey bee, Apis mellifera nutritionally changing the DNA methylation levels contributes to the oncogenic establishment of substitute castes [21,71].

All this supports the hypothesis that caste development and sociality evolution are connected with the DNA methylation, and recent actual evidence from the social insects like *Hymenoptera* (ants, wasps, and bees) reveals that this relationship is not general [72–76]. tRNA aspartic acid methyltransferase 1 (TRDMT1), most usually recognized as DNMT2, is the noncanonical member of the DNMT family (an enzyme that works in DNA methylation) known to methylate the tRNA, not DNA [77,78], and it is usually considered that these functions are preserved in insects [79]. This hypothesis is underpinned by the opinion that in *D. melanogaster,* the nonappearance of DNMT1 and DNMT3 is related to the damage or else dangerous drop of DNA methylation [80].

## DNA methylation is absent in Diptera due to lost DNMT1 and DNMT3

DNA methylation is based on the two types of DNMT1 and DNMT3 methylates in mammals, while in insects, these methyltransferases are absent due to the great limitation toward DNA methylation as in *D. melanogaster* [81]. But in contrast, *Hemiptera*, *Coleoptera*, and *Lepidoptera* lack DNMT3, but DNA methylation is observed in the absence of DNMT3 as DNA methylation is not conserved in insect than mammals as in *Hemiptera, Coleoptera, Lepidoptera*, silk moth, and many others.

In distinction to mammals, theDNMT toolset of insects displays considerable points that in their genomes, DNA methylation is not preserved (as mention above), i.e. Bombyx mori and silk moth have verifiable firm DNA methylation in its genome but copies of DNMT3 are absent [70,82]. In insects, functional methylation systems can be realized in the absence of DNMT3. Loss in altered lineages but the frequency of DNMT3 is unidentified due to the absence of widespread relative data. Ten-eleven Translocation (TET) (enzymes that provided a mechanistic foundation for a frequently imaginary pathway for DNA demethylation) in the honey bee newly shows that the sole Tet enzyme is the competent of changing 5mC to 5hmC [83]. In insects, Tet enzymes could exhibit handy promiscuity, as 5mC demethylation and N6-methyladenine demethylation were observed due to the Tet enzyme homolog in DNA and mRNA of *D. melanogaster* [84,85]. In insects, the Tet enzyme dispersal and its association with the occurrence of 5mC are presently unknown.

## DNA methylation in contrast to mammals and CpG methylation differ in insects from mammals

DNA methylation differs significantly in insects from mammals due to different regulatory functions. The percentage of methyl cytosine varies in eukaryotes as it is 0–3% in insects and 5% in mammals. In mammals, 60%-90% CpGs are methylated in the whole genome and the DNA methylation is well characterized in them as compared to the insects [86]. CpG dinucleotides are heavily methylated in mammals except for the CpG Islands in which promoter regions that overlap are unmethylated. In invertebrates including insects, DNA methylation at the CpG sites is almost negligible and linked to the gene bodies (transcribed part of the gene) [87]. In *Drosophila melanogaster*, there are two proteins that encode for DNA methylation and resemble cytosine DNA methyltransferases and a mammalian methyl-CpG-binding domain (MBD) protein [88].

By doing the profiling of methylation in insects, we come to know that methylation typically representing Hymenoptera and to a minor degree Coleoptera and Lepidoptera exposed mostly alike designs of DNA methylation, which mainly aims to exons of protein-coding genes [72,74–76,82,89–92]. Through different experiments on different insects, we came to know that DNA methylation-targeted genes were universally disclosed among several kinds of tissues, between diverse transforms in ants [72,75], and between progressive phases in *the Nasonia vitripennis*, parasitoid wasp [90].

A majority of gene ontology explanations disclosed that most genes assist in simple cellular functions and reveal a significantly methylated state between species, and at the sequence level, they are extremely preserved [89–94]. Through different research studies, different findings are obtained, which strongly suggest that in insect genomes, DNA methylation is not random, but a concrete clarification for this opinion remains indefinable [87].

## DNA methylation role in social connection

DNA methylation has a great influence on the social behavior of insects. Honey bees are the best example for their social behavior, memory formation, and age-related behavior for working due to DNA methylation. Queens have a lower level of DNA methylation as compared to workers. So, inhibition or silencing of DNA methylation in newly hatched embryos produces queens rather than workers [71].

## Role in gene expression or gene silencing

DNA methylation has both repressive and expressive effects, as in the case of honey bees, lower production of DNA methyltransferases (DNMTs) affects the social behavior by showing the repressive effect, while in other insects, a higher level of DNMTs causes higher epigenetic modifications due to the higher level of DNA methylation [87].

## Importance of DNA methyltransferase 1 and posttranslational modification, as predictive

The level of DNA methylation can be estimated by the presence or absence of DNA methyltransferases and normalized CpG dinucleotide content. DNMT1 is much wider than DNMT3. So, DNMT1 helps in the prediction of DNA methylation [87].

### (a) In insects, the role of DNA methylation awareness

Evidence in insects recommends gene bodies DNA methylation preform part in the maintenance of transcript integrity, and also presented as the evidence that mRNA starts or splices outline are influenced by it and might show a particular part in it [88,92,95–99]. In mammalian studies, we came to know the idea that DNA methylation in gene might control substitute intragenic promoters and disturbing alternative transcription [88,100].

A hypothesis is given that de novo DNA methylation is a significant part in developing responsiveness to natural features and the arrangement of growing plasticity in honeybee Ap. mellifera possible case [21,101,102]. Through epigenetic evidence, we know that during the sequence of organismal growth, the freshly presented difference in DNA methylation might point to changes in the direction of gene transcription, which might improve progressive plasticity and deliver a significant mechanism by stimuli of environment and responsiveness [14].

## DNA methylation in plants

Plants are the most influential system to study DNA methylation. DNA methylation’s outcomes and associations are context-dependent. DNA methylation has been judged by various techniques. The production of DNA methylation resolution maps through the entire genome are possible by Whole Genome bisulfite sequencing (WGBS). It has been used to sequence an increasing number of plant methylomes ranging from a model plant *A. thaliana* to economical crops like Z. mays. DNA methylation in plants methylates DNA in three different context: dinucleotide CpG or CG and trinucleotide CHG and CHH sites (C, T and H = A) [103,104]. CpG and CHG sites are symmetrical, and CHH sites are asymmetrical. In plants, epigenetic mechanisms indicate that environmental conditions alter the DNA methylation position [105]. It is to be noted that in coding regions and promoter region are more methylated than actively transcribed sequences in plants [106], showing that DNA methylation regulates gene expression [107]. DNA methylation reassures the wrapping of DNA into the so-called heterochromatin-imposed silent state, for example, by constructing the DNA reserved to transcription activators in plants [108].

DNA methyltransferases (DNMTs) are working as a generator of DNA methylation in plants, and methyl groups are enzymatically transferred from *S-adenosyl methionine* (SAM) to cytosine [109]. Small interfering RNAs (siRNAs) are controlling the DNA methylation in plants, This RNA-directed DNA methylation (RdDM) is referred by two corresponding pathways, controlling initiation and establishment of DNA methylation in every sequence [110,111].

There are a significant group of enzymes that assist plant-specific DNA methylation, known as CHROMOMETHYLASES (CMTs), which are characterized by the presence of a chromatin organization modifier (CHROMO) domain between the cytosine methyltransferase catalytic motifs I and IV [112]. The highest levels of DNA methylation are typically found in centromeres and peri-centromeric regions in plants [113,114]. There are five different classes of methylated genes in flowering plants:

(a) UM (Unmethylated)

(b) gbM (gene body methylated)

(c) TSS (transcriptional start and termination site)

(d) Mcg/mcgh (methylated CG or methylated CGH)

(e) CHH/RdDM. In plants, generally, high DNA methylation occurs in these transposable elements [104,113,115,116]. Using recent studies in the model plant Arabidopsis thaliana, we examine the biological importance of DNA methylation, as well as demethylation, in plant immunity against nonviral pathogens.

### (a) DNA Methylation in Arabidopsis

DNA methylation targets tandem, and dispersed repeats generally result in transposable elements [117,118]. At these loci, DNA methylation occurs in three diverse sequence contexts: symmetrical CG dinucleotides (usually highly methylated at 80–100%); symmetrical CHG, where it resembles A, T, or C (methylated at 20–100%); and asymmetrical CHH (usually slowly methylated at 10% or less) [114,119]. DNA methylation can also be present at gene bodies, exclusively at CG residues. In this particular case, DNA methylation function is at present unclear and hence the focus on repeat-associated DNA methylation in promoters and introns (Table 1) [120].

**Table 1.** Summary of arabidopsis genome DNA methyltransferase [187]

Family Mammalian and Arabidopsis	Arabidopsis genes	Function	Primary effects on DNA methylation	Effect on H3K9Me
DNMT1; MET Dnmt2 Dnmt4, DRMs Not known in mammals; Chromomethylase	MET1 METIIa METIIb METIII At5g25480 DRM1 and DRM2 CMT1 CMT2 CMT3	Maintenance of CG methylation ? ? ? ? De novo CG and non-CG Methylation and RdDM ? ? Locus-specific maintenance of non-CG methylation	Loss of CG methylation ? ? ? ? Loss of non-CG methylation at specific loci such as FWA and SUPERMAN ? ? Loss of CNG methylation pericentromeric repeats and at specific loci	Loss of H3K9Me ? ? ? ? No effect ? ? No effect

## DNA methylation in mammals

As in other organisms, the cytosine methylation occurs in mammals too and enzymes DNA methyltransferase (DNMT) and ten eleven translocation (TET) are involved in it. In mammals, DNA methylation plays its role in silencing of transposable elements, X-chromosome inactivation, regulation of gene expression, and genomic simprinting. In mammalian DNA, 70%–80% somatic tissue DNA is methylated at all CpG positions, sequences that have high methylation include exons of genes, nonrepetitive intergenic DNA, repetitive elements (including transposons and their inert relics), and satellite DNAs, and researchers show that the CpG dinucleotides have more frequency of methylation [121]. DNA methylation is important for the proper placenta and embryo development [122]. For normal placenta development, DNMT is important (DNMT10) [123]. Loss of dnmt10 results in placenta dysmorphology in mice [124,125]. In comparison with other tissues, placentas of mouse and humans are hypomethylated [126–128]. On a symbolic set of mammalian placentas by performing Methy l C-seq, high methylation is found in single genes rather than an complete group of genes, for example, in placentas with PMD (partially methylated domain)/ HMD (highly methylated domain).

## De novo DNA methylation mechanism and enzyme

In mammals, the establishment of DNA methylation patterns occurs through embryonic growth by the de novo DNA methylating enzymes known as Dnmt3a and Dnmt3b. These enzymes are conserved by the Dnmt-1-mediated process during cell division, and epigenetic markers are based on DNA methylation patterns of the genome of an organism during cell division. It is the reason that DNA methylation tells us about archetypal mechanism of epigenetic inheritance [71].

## Developmental period

DNA methylation is necessary for mammalian’s development as addition of methyl groups to the 5-position of cytosine changes the structure of the DNA groove to which DNA binding protein binds. Methylation of CpG promoters is used to prevent transcriptional initiation and makes sure the gene silencing and parasitic DNAs. CpG poor promoters are less efficient for a tissue-specific gene expression maintenance [129]. Mammalian genome is greatly CpG-depleted, and 60%-80% human genome is methylated out of 28 million CpGs [130].

## DNA methylation and diseases

Aberrant DNA methylation is a possible cause of various health problems in cloned organisms [130]. Aberrant hypermethylation CpG Islands are observed in human’s colorectal tumors that are due to upregulation of DNA tranferases, changes in DNA methylation occur in a developmental stage, and tissue-specific gene’s DNA methylation often leads to tumor development in the form of CpG Island hypermethylation [131]. Loss of de novo DNA methylation can be beneficial to tumor progression as it is linked to cancer due to hypermethylation [132].

## Pathological diseases

Concernments in epigenetic factors (DNA methylation, histone modification, and genomic imprinting) are the cause of developing many diseases such as diabetes, cardiovascular diseases, and cancer. Oxidative stress is related to pathophysiology of many diseases referred to high synthesis of reactive oxygen species (ROS). Oxidative stress causes the limitation of antioxidant defenses, which is responsible for their metabolism and causes an equilibrium state between production and removal [133].

### (a) DNA Methylation in humans

Keeping in mind the global Sustainable Development goals, ensuring healthy lives and promoting well-being for all ages and every station are focused [22]. In human, DNA CpG might be alternating but is extremely methylated, and segments containing GC bases more than 50% are known as the CpG Island (CGI) and roughly 200–300 bp long [134,135]. DNA methyltransferases (DNMTs) catalyze the methylation reactions in humans, and enzymes DNMT1, DNMT3a, and DNMT3b catalyze the addition of methyl groups mostly at the CpG dinucleotide [136]. In humans during early embryogenesis, DNA methyltransferases (DNMTs) 1, 3a, and 3b are the enzymes that establish methylation patterns.

Abnormal DNA methylation is also related to several diseases [137], which include cancer [138–140], autoimmune diseases, rheumatoid arthritis [139,141], diabetes [142,143], and neurodegenerative diseases such as Parkinson's and Alzheimer’s[144–146]. Several new results show that psychological factors such as aggression, anxiety, happiness, sadness, and the life satisfaction also initiate abnormal patterns of DNA methylation and results lead to numerous mental diseases [147–150]. Epigenome-wide association (EWA) studies have ever found a durable link among methylation and psychological exposures in humans [151].

### (b) DNA methylation in cancer in humans

The earliest proof about DNA methylation involved in carcinogenesis directly came in 1994 when Herman et al. disclosed that tumor suppressor gene VHL may be silenced by hypermethylation of the VHL gene promoter in some renal carcinoma cases [152]. Abnormal DNA methylation of fixed loci has been observed in many types of cancers such as colon, breast, ovarian, esophagus, bladder, and bone cancer [153].

### (c) DNA methylation in early cancer diagnosis

When DNA methylation is deregulated, it frequently interrupts signal pathways, which contribute to the growth of numerous sicknesses, and all these are initial epigenetic variations for tumor identification [154].

In several tumor cells, irregular DNA methylation has been examined, in cells like cells of the colon, breast, cervical, and ovarian cancer, which are connected with acknowledged tumor or oncogene suppressor genes' appearance. This is why in malignancy diagnosis, DNA methylation was used as a biomarker [155].

### (d) DNA methylation biomarker in cancer

DNA methylation is not only related to cancer but also to levels of mRNA. Barrett’s esophagus (BE) was checked in 195 patients who had passed methylation, 125 times higher danger of changing into esophageal adenocarcinoma than in healthy and controlled genes like CDH13, TAC1,NELL1, AKAP12, p16, RUNX3, HPP1, and SST, which individually were initiated to anchorage methylated sites [156].

In ESR1, promoters and 14-3-3-sigma methylation status were diverse among healthy people and patients with breast cancer [157]. Also, research showed that tissue factor pathway inhibitor 2 (TFPI2) methylation was noticeable in colorectal cancers of human [158]. Cancer is helped by not only abnormal promoter methylation, which deregulated tumor-related gene expression [155], but also biomarkers for different types of cancers like pan cancer [159], prostate cancer [160], ovarian cancer [161], and bladder cancer [162].

### (e) Barriers in applying DNA methylation biomarkers

In spite of the potential of biomarkers, quite a little biomarkers attained the probability for acceptance in clinic; in malignant tumor, the most applicant biomarkers are elaborated; as with disease phenotypes, approaches used in gaging DNA methylation need to be corrected and be reproducible; and DNA methylation must be delicate and fixed and also have high analytics in cancer [155].

### (f) Nutrition and its role in DNA methylation in human

Nutrition has a significant part in vigor, sickness circumstances, and impacts in utero growth, DNA has the areas that are delicate to methylation built on little earlier reviews on nutrition, and these areas are recognized as methylation variable positions (MVPs); MVP observation and explanation showed that for part of nutrition in the fetal origin of mature diseases, several theories are put forward to clarify the fetal origin of adult diseases [163]. Different theories define that nutrition has a significant part in adult disease danger and transmission to the progeny [164]. Fetal growth is related to insulin resistance, and diabetes and adult cardiovascular disease are also expected that they will affect impairment in them [165,166].

Through an earlier study, I was exposed that prenatal and initial postnatal experience to the shortage of food might upsurge the danger of fatness [167], lung diseases, schizophrenia, and also women breast cancer [168]. Methionine and folic acid in humans have a vital part in DNA modifications and in pyrimidine and purine synthesis, and folic acid is known for roles in providing one-carbon breakdown that delivers carbon to them. In utero and in adults, it is vital to have a decent comprehension of its part in DNA modifications [163].

DNA methylation is increased by periconceptional additions of folic acid up to 4.5% of IGF2 [169]. Homocysteine and folic acid status are inversely related to each other, these were shown by different studies of cord blood sample analysis, and they are vice versa of each other [170].

## Nutritional values for fetal development

Development of a zygote to an individual or whole organism is maintained by the series of cellular processes. DNA methylation causes certain epigenetic modifications during DNA methylation, which is necessary for mammalian development [130]. Nutrition at different stages of life can affect the epigenetic gene function. High consumption of methionine causes the DNA methylation and deregulation of the gene expression [171].

## Limits or short comings of DNA methylation

DNA methylation is a time-consuming and relatively expensive process. Detection, which is highly dependent on assay conditions and DNA quantitation, assesses methylation status and CpG residues, and we cannot determine differences in methylation between different CpGs contained in primer recognition sequences.

It requires large amounts of DNA and lengthy procedure and only assesses methylation status of CpG residues at specific DNA restriction sites for detection.

Complexity of PCR-based measurement of methylation and problems in primers with respect to different sites of methylation, and also, we cannot be sure that either the require region is amplified or not and the primer and other condition are working optimally, if so, after that, the Qualification and Quantification of Methylation is a lengthy procedure.

## Future prospective of DNA methylation

Epigenetics is a unique and successful research. Today, many queries related to epigenetics are unanswered, but there is hope that they will be attempted tomorrow. The unique features of epigenetics m,ade it useful and necessary for targeted therapeutics, pathogenic insight, transplantation, and biomarker discovery.

As a biomarker DNA methylation is not that delicate technique to tissue handling in comparison with proteins and RNA because it can be achieved even from little amount of DNA isolated from fixed tissues [172]. Future education must involve a balanced method by examining the DNA methylome in a genome-wide way to offer an extensive and more detailed study.

Nutrition has significant part in health, utero development and also has an impact on disease conditions. There are some areas on DNA, known as methylation variable positions (MVPs), and these regions are delicate to methylation based on a few earlier studies on diet. Some theories revealed that nutrition plays a role in adult disease risk and also its transfer to offsprings [164]. It is known that folic acid and methionine play an important role in DNA modification, so it is important to understand the role of folic acid in both utero and adult. The DNA methylation is increased by 4.5% of IGF2 due to periconceptional supplementation of folic acid [169]. DNA methylation of 12 genes (AMN,BDH2, C9 or f64, FBN3, EIF2C3, MGC33486, PDE2A, PVRL2, RUNX1T1, ZBTB11, ZPBP2, and ZNF187) is directly connected to methylation of five genes (ATP5F1, BMX, MDS032, FSTL3. and CYP26C1), and homocysteine levels displayed an opposite connection with the Homocysteine level [173]; why it happened?

The layering and conversation of different kinds of epigenetic material is another stimulating and unfamiliar direction for future education in insects. DNA methylation contains the relations between the large set of proteins in vertebrates and fungi [174], such as those related to histone modification systems, and has to be understood more [175–177]. DNMTs and MBPs play a role in the recruitment of histone modification proteins [178–183] and the proteins responsible for the transformation of chromatin in the mammals [181,184]; and understanding genes and their working is required with more knowledge.

The global genomic methylation studies in mammalian genomes, especially in genomes of human, are renewing the idea that DNA methylation play its role in development and differentiation that require more research and that has to be solved and understood. The role of anomalous DNA methylation in cancer has been credibly discussed [179], but still more information and research areneeded on it; in the future, we might be able to prevent cancer and will also cure it. Various other human diseases are also linked with abnormalities in DNA methylation [185].

**Graph.2;:**
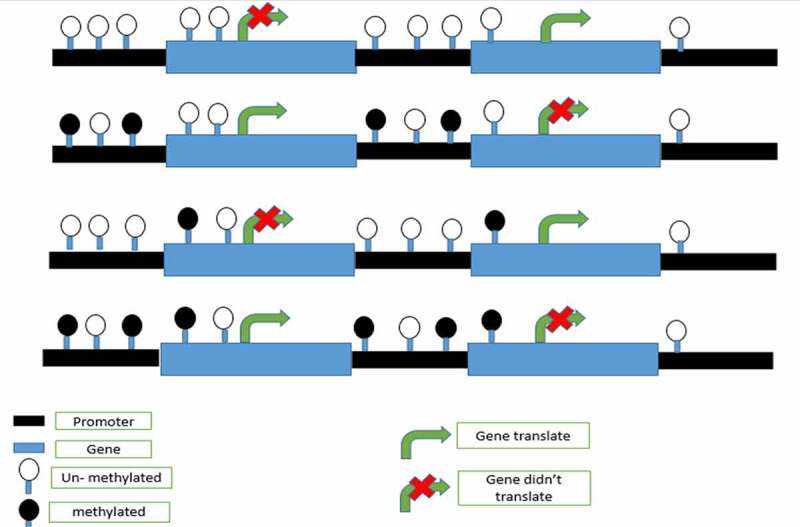
*In the graph, it is shown that DNA methylation on any part of gene may stop translation or may not depend on the DNA* methylation role.

**Graph.3;:**
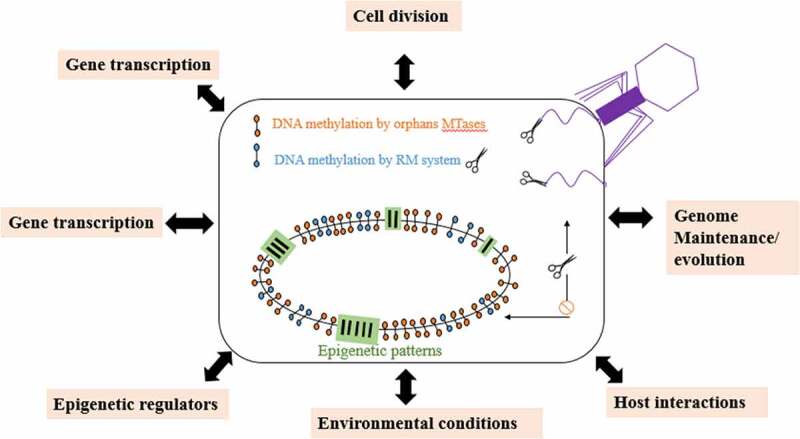
Dfferent function performed by DNA methylation in bacteria and archaea.

**Graph.4;:**
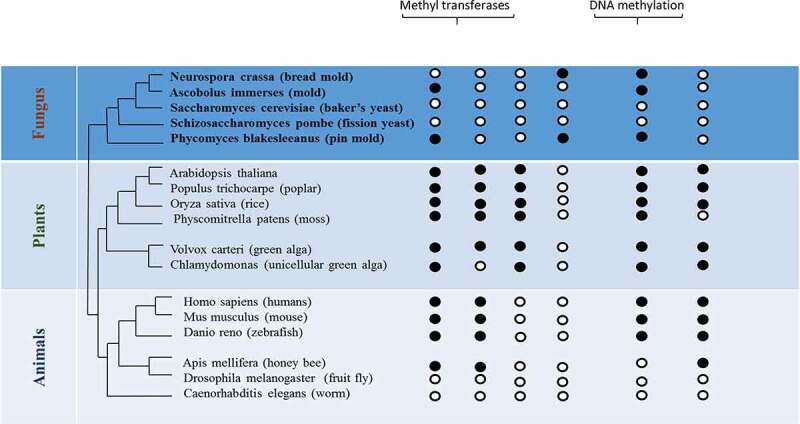
The diagram explains how the DNA methylation is distributed and the presence of DNA methylatransferase across the eukaryotes. The black and white circles indicate the presence and absence of DNA methyltransferase/methylation, respectively [186].

**Graph.5;:**
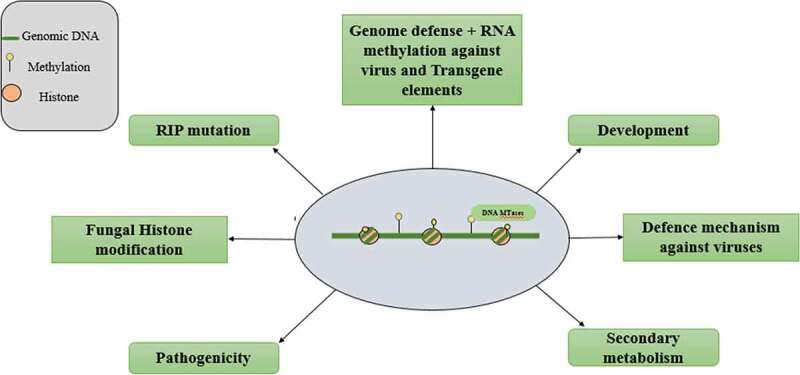
Different functions performed by DNA methylation in fungi.

**Graph.6;:**
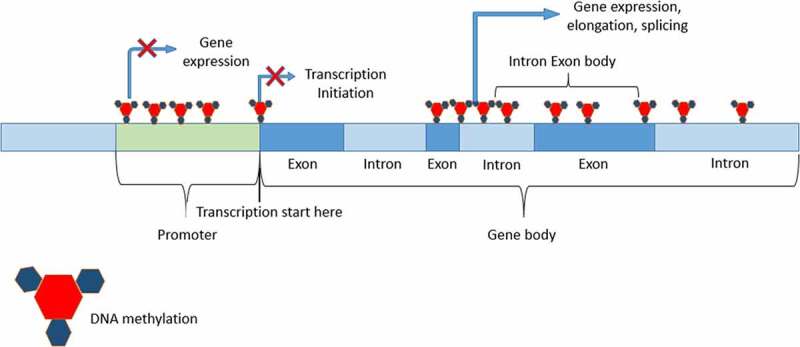
The effect of DNA methylation on different sites of DNA.

**Graph.7;:**
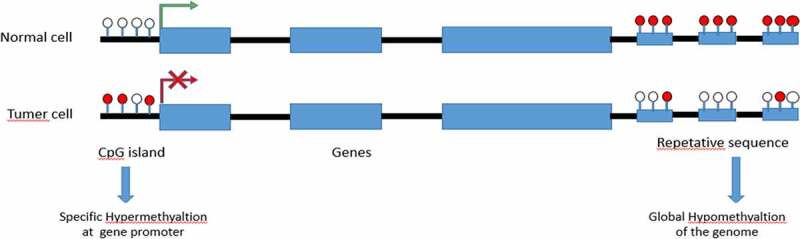
The graph shows the effect of methylation on tumor.

## Conclusion

As goals be global Sustainable Development we try to focus on those goals like health in which bacteria as disease causing agent, fungi rule in disease and also in environment and plants are also focused [22]. By going through all of these, we came to the conclusion that DNA methylation is a very important thing in the tree of life. Starting from simple organism like bacteria, it plays its role in their genome and also perform functions like saving the bacteria attached to its own restriction enzymes (RE), which act against the viral phage genome and other agents. Also as camper to other eukaryotes, DNA methylation is present in the noncoding region and in the coding region and it is absent and gives rise to consensus that DNA methylation is initially dedicated to transposable elements (TEs), which are supposed to maintain the integrity of DNA, and also that different levels of methylation are present in it and lack the gene body methylation. In the insects, methylation of the gene body was considered and does not have such a good modal organism for study due to which lack of information regarding the DNA methylation in insects is present; in plants, it is also present in different areas of its genome and has different functions and also is the case with humans in which they play a role in cancer and other diseases related to genome. As the area of genomics will gain more information and more advanced techniques will come onward, the function and role of DNA methylation will be clearer and will be used for the betterment of life and betterment of nature.

## Data Availability

Books, research articles, and internet
